# Perspectives on user engagement of satellite Earth observation for water quality management^☆^

**DOI:** 10.1016/j.techfore.2023.122357

**Published:** 2023-04

**Authors:** Lara Agnoli, Erin Urquhart, Nikolaos Georgantzis, Blake Schaeffer, Richard Simmons, Bilqis Hoque, Merrie Beth Neely, Claire Neil, Jacques Oliver, Andrew Tyler

**Affiliations:** a School of Wine & Spirits Business, Burgundy School of Business, Université Bourgogne Franche-Comté, CEREN, EA 7477, 29 Rue Sambin, Dijon 21000, France; b Science Systems and Applications, Inc., Ocean Ecology Laboratory, NASA Goddard Space Flight Center, 8800 Greenbelt Rd, Greenbelt 20771, MD, USA; c U.S. Environmental Protection Agency, Office of Research and Development, 109 TW Alexander Dr, Durham 27709, NC, USA; d Faculty of Social Science, University of Stirling, Stirling FK9 4LA, Scotland, United Kingdom of Great Britain and Northern Ireland; e Environment and Population Research Centre, 1, 7 Block #E, Dhaka 1207, Bangladesh; f Global Science and Technology, Inc., 7501 Greenway Center Dr. #1100, Greenbelt 20770, MD, USA; g Scottish Environment Protection Agency, The Castle Business Park, Strathallan House, Stirling FK9 4TZ, Scotland, United Kingdom of Great Britain and Northern Ireland; h U.S. Environmental Protection Agency, Office of Water, Office of Science and Technology, 1200 Pennsylvania Avenue NW, Washington, DC 20004, USA; i Scotland’s International Environment Centre, Faculty of Social Science, University of Stirling, Stirling FK9 4LA, Scotland, United Kingdom of Great Britain and Northern Ireland

**Keywords:** Water quality, Earth observation, Satellite remote sensing, Knowledge transfer, Technology transfer, Data accessibility

## Abstract

The management and governance of our surface waters is core to life and prosperity on our planet. However, monitoring data are not available to many potential users and the disparate nature of water bodies makes consistent monitoring across so many systems difficult. While satellite Earth observation (EO) offers solutions, there are numerous challenges that limit the use of satellite EO for water monitoring. To understand the perceptions of using satellite EO for water quality monitoring, a survey was conducted within academia and the water quality management sector. Study objectives were to assess community understanding of satellite EO water quality data, identify barriers in the adoption of satellite EO data, and analyse trust in satellite EO data. Most (40 %) participants were beginners with little understanding of satellite EO. Participants indicated problems with satellite EO data accessibility (31 %) and interpretability (26 %). Results showed a high level of trust with satellite EO data and higher trust with in-situ EO data. This study highlighted the gap between water science, applied social science, and policy. A transdisciplinary approach to managing water resources is needed to bridge water disciplines and take a key role in areas such as social issues, knowledge brokering, and translation.

## 1. Introduction

The effective management and governance of our surface waters is core to life and prosperity on our planet, including food and energy production, the conservation and promotion of biodiversity, and the enhancement of natural capital to sequester and store carbon. It also impacts our economic capacity and wellbeing and reflects the societal challenges of unequal access shaped by age, gender, and other socioeconomic factors. The United Nations’ (UN) sustainable development goal (SDG) 6 is the provision of clean, safe, sustainable water and sanitation. Other UN SDGs supported by clean water focus on reducing poverty and ending hunger (SDG 1 and 2); sustaining health and wellbeing (SDG 3); and economic development (SDGs 8, 9, 11, and 12) (MEA, 2005; Völker and Kistemann, 2011 ). Declines in the quantity and quality of water are a pre-eminent risk to society and the global economy (COM, 2012; World Economic Forum, 2015), particularly in developing countries with low resilience, inadequate sanitation, poor health provision and weak governance (Friel et al., 2008). Yet despite our dependencies on the numerous ecosystem services provided by water, our surface waters face multiple and compounding pressures from changes in land use and climate change, nutrient enrichment, brownification, and other natural and anthropogenic driven environmental perturbations (IPCC, 2012; MEA, 2005; Ormerod et al., 2010).

Ostrom’s (1990) influential studies of common-pool resources, including water, resulted in an important set of design principles for effective governance at the local level, with adaptations to consider key contextual differences at the global level (Stern, 2011). At each level of scale, a key design principle involves “monitoring of the common-pool resource and its use that is accountable to the interested and affected parties” (Stern, 2011). For example, empirical and experimental research on the electricity sector (Delmas et al., 2013; Martín et al., 2016), has shown that efficient monitoring and frequent feedback on individual and peer behavior reduce over-consumption. Moreover, Moxnes (2003) has shown that, even in the absence of tragedy of the commons considerations, measurement accuracy and inclusion of both recent and past feedback improve the management of complex and uncertain systems.

Applying this logic to water management, frequent and accurate monitoring of water quality and quantity may also be an efficient way of inducing more responsible use of the resource and a better understanding of the consequences of one’s own actions on the stock and the quality of water resources. Water managers and socioeconomic policymakers deal with risk and uncertainty (World Water Assessment Programme, 2012); the more these uncertainties and risks are understood, the more effectively managers and policymakers may plan, design, and manage water systems to reduce risks. There is, therefore, a fundamental need to monitor our waters at scales that enable effective management to mitigate these pressures, yet there are numerous regions of our planet that have little or no data to support water governance (COM, 2012; MEA, 2005; Volker and Kistemann, 2011; World Economic Forum, 2015). Even regions of the globe with extensive monitoring and data are temporally and spatially patchy at best.

In response, the last ten years have seen a rapid growth in the exploitation of satellite Earth observation (EO) technologies for monitoring water quality (Tyler et al., 2016), and of algorithmic solutions to dealing with the optical complexity of inland and coastal waters for retrieving reliable and useful data (Neil et al., 2019; Pahlevan et al., 2020; Schaeffer et al., 2015; Spyrakos et al., 2018).

In this way, linking satellite EO (observations derived from satellite remote sensing missions) with existing in-situ EO (measurements from discrete water samples or electronic sensors in the environment) and models (UNDP, 2006) enables proper water resources management, planning, design and operation (El Serafy et al., 2021). Briefly, operational satellite examples include the European Space Agency’s Copernicus program (Berger et al., 2012) with Sentinel-2 MultiSpectral Instruments and Sentinel-3 Ocean and Land Colour Instruments; and the National Aeronautics and Space Administration/U.S. Geological Survey Landsat missions (Loveland and Dwyer, 2012). These satellites allow us to monitor our waters at scales that enable effective management to mitigate these human pressures. These satellites also allow for long-term investments and benefits that can cover the full technology adoption lifecycle (Hillmer, 2009). Based on a technology adoption lifecycle, the water management community is in the early adopter phase and transitioning to the early majority phase (Rogers, 2003). Satellite derived water quality information such as chlorophyll *a*, cyanobacteria biomass, colored dissolved organic matter, and total suspended sediments are now at the phase where there are some early adopters of the technology and progress is accelerating quickly. The COVID-19 pandemic may accelerate use of satellite and other monitoring technologies, such as big data analytics, to oversee the water sector while alleviating issues caused by social distancing requirements and travel restrictions (Brem et al., 2021; Renukappa et al., 2021). There is now a real opportunity for data acquired through satellite EO to become widely used through the development of global partnerships (SDG 17), enabling the provision of information across a suite of valuable indicators of water quality and ecosystem conditions.

If collected at appropriate scales and communicated effectively, such data may deliver new knowledge, create shared understanding, and democratize the debates at community, national, and international scales to drive change in sustainable water management. These data provide critical opportunities to drive the changes in behavior and governance needed to combat the effects of climate and the legacy of poor water management. More broadly, EO business opportunity valuation has been estimated at $66 billion in 2020 (LSE, 2018), and is projected to double by 2030. In the water quality sector, the annual avoided costs for satellite measures of chlorophyll-a in U.S. lakes and reservoirs with Sentinel-3 were $5.7 ± 1.59 million and $42 ± 9.5 million for Landsat 8 (Papenfus et al., 2020). Another demonstration of satellite water quality sector valuation was the availability of these data-yielded socioeconomic benefits by improving human health outcomes valued at approximately $370,000 for a single recreational advisory at Utah Lake, UT, in 2017 (Stroming et al., 2020).

Despite this progress and opportunity, the uptake of satellite EO technologies for water related decisions remains limited (Schaeffer et al., 2013). To some extent, the gradual acceptance of many new technologies is common. The evolution of satellites for meteorological measures followed a similar path; once satellite-derived measures were effectively demonstrated, satellite data became a routine expectation of stakeholders (Schmetz and Menzel, 2015). There is growing awareness within the community of a lack of capacity to fully realise the potential and market opportunity of satellite EO technologies (Sadlier et al., 2018). This lack of capacity includes the lack of expertise to understand the opportunity and value of EO data across sectors, which includes challenges in accessing data and the time and cost of processing it. Anecdotal evidence from practitioners highlights the challenges in recruiting a suitably skilled workforce as demand for skilled individuals exceeds supply in a rapidly expanding market of opportunity. This is resulting in a latency in the confidence in the new technology (EO), and an overall lack of concerted effort to support satellite validation and integration. So far, such challenges are preventing the full and proper use of satellite EO for water quality monitoring.

As demonstrated with the meteorological satellites, it is important that new technologies represent ‘responsible innovation’ (Stilgoe et al., 2013), taking account of the extent to which social as well as technical value is embedded in innovations (Guston and Sarewitz, 2002). This requires constructive technology assessment, including dialogue between innovators and users of technology, to ‘articulate the demand-side of technology development’ (Schot and Rip, 1997) and uncover any ethical and moral dilemmas inherent in these innovations (Joss and Bellucci, 2002). In summary, the technical, economic, and societal case for satellite technologies to support the monitoring and governance of water resources is strong and well known to the research and innovation community responsible for the development of this capability, including the international Group on Earth Observation (GEO) AquaWatch community (https://www.geoaquawatch.org). However, questions remain around the perceived needs and barriers to implementation at national, regional, and local levels around the world.

Here we explore the perceptions of opportunity and barriers to exploiting this satellite EO capability with the potential user community around the globe. In response to this gap in knowledge, the GEO AquaWatch Initiative prepared a survey targeted toward individuals working in the water sectors including governance, industry, academia, and any other relevant stakeholders. The survey was one of the first of its kind in the water sector specific to satellite water quality. To place the current study into the context of similar efforts we did a brief Web of Science search using the keywords stakeholder, perception and a hierarchy of narrowing areas from broad science and data topics toward a specific satellite water quality topic.

**Fig. 1.**
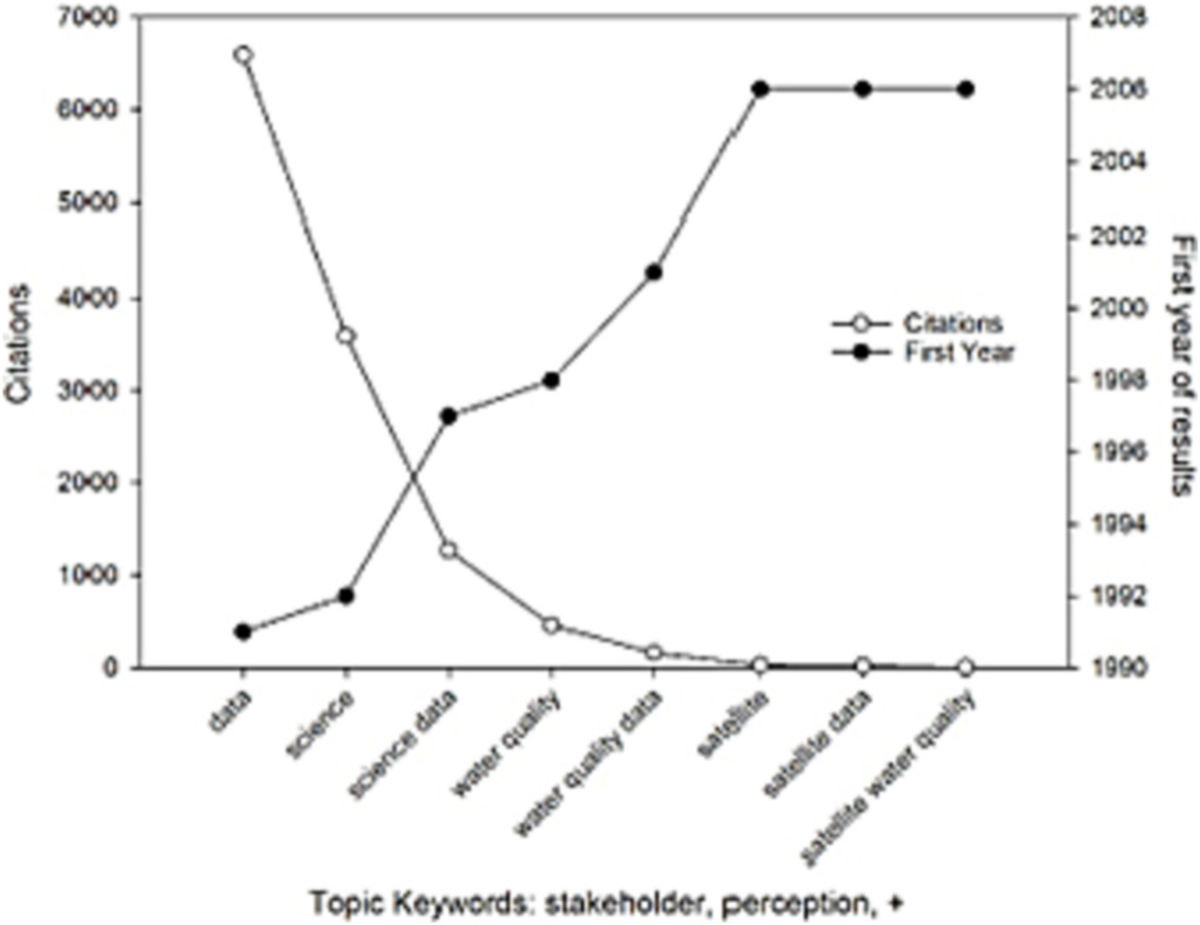
Stakeholder perception studies related to satellite water quality data.

Fig. 1 indicates stakeholder perception of broad topics related to data and science are numerous and become available in the early 1990s, where more specific focus on water quality and water quality data are below 500 results and not available until the late 1990s. Stakeholder perceptions regarding satellite and satellite data drop to 32 results and not available until 2006. Finally, stakeholder perception studies related to satellite water quality only have 4 results since 2006.

To explore and develop a roadmap to deliver usable and accessible satellite EO products that better meet the needs of the water sector specifically, and to serve communities and national statutory requirements, the current study thus provides novel insights from a knowledgeable group as a useful ‘can opener’ for further discussion and future research (Carter et al., 1991). Research objectives (RO) were: (RO1) to assess knowledge and attitudes toward understanding of satellite based EO derived water quality data products across the water sector; (RO2) identify barriers and problems in the adoption of satellite based, EO-derived water quality data products; and (RO3) analyse trust in satellite-based, EO-derived water quality data products. Overall, the aim is to gain an understanding of capacity building requirements for both developed and developing nations. The data will be used to support targeted investment to advance this capacity with partner countries to improve water quality.

## 2. Methodology

The survey was designed for individuals working in water sectors including academia, industry, those responsible for governance, and any other relevant stakeholders including statutory and societal. To meet the research objectives, an ad-hoc survey was built to collect information on 1) participant characteristics, 2) participant knowledge, 3) attitudes toward satellite-based, EO-derived water quality data, and 4) barriers and problems.

Table 1 details the information collected through the survey and predefined multiple-choice options including binary, nominal, and ordinal variables. Introductory text directed participants to examples of satellite EO capability for monitoring lakes found on the GloboLakes portal to provide context. At the time of the survey, this demonstrated data for 1000 lakes globally from the United Kingdom’s Natural Environment Research Council project GloboLakes (http://www.globolakes.ac.uk). The survey was developed and tested within each of the partner host institutions to assess the robustness of the questions, clarity of phrasing and overall timing. The aim was to keep the survey to <10 min and completion times averaged 5 to 8 min. The final version was first translated into English, German, Chinese, Spanish, and French by GEO AquaWatch members. The survey was deployed within our GEO network through SurveyMonkey.com where participants chose their preferred language from the list of options. Data were collected by adopting convenience and snowball sampling techniques (Dusek et al., 2015). Social media and the networks connected to the GEO AquaWatch Initiative (https://www.geoaquawatch.org) were used to deploy the survey globally. This included the distribution of the questionnaire through research networks as well as through partner organizations including NOAA, NASA, and intergovernmental organizations such as the United Nations Environment Programme GEMS/Water and World Water Quality Alliance. The ambition was to garner a cross sector sample a global population of practitioners. The questionnaire was made available for one calendar month to allow for distribution and engagement in March–April 2019.

We used Stata14 and IBM SPSS Statistics19 software to analyse the data. Univariate and bivariate analysis techniques were applied. The participants’ knowledge and attitudes toward EO water quality data products (RO1) and problems in adopting these technologies (RO2) were explored through univariate data analysis techniques, and absolute and relative frequencies of the analysed information, together with mean and standard deviations (Carifio and Perla, 2007; de Winter and Dodou, 2010). Trust (RO3) in satellite EO water quality data products and their determinants and implications were pursued applying univariate and bivariate data analysis techniques (Mulder et al., 2006). After exploring the attitudinal traits linked to the use of EO water quality data products - namely knowledge, usefulness, trust, and problem perception - correlations analyses were performed to highlight relations among attitudinal traits. Given the ordinal nature of the analysed variables, the nonparametric Spearman’s correlation test was applied to measure the strength of monotonic relations among them (Myers and Sirois, 2004). The resulting coefficients can range from −1 to +1, namely from no correlation (when ρ = 0), to perfect monotonic positive (ρ = +1) or negative (ρ = −1) correlation.

Based on correlations among constructs, an attitudinal model was built to explain determinants and implications of trust in satellite EO water quality data products. Given the ordinal nature of the analysed variables, Ordinal Logistic Regressions (Brant, 1990) was used to test relations among attitude traits in the model (Supplementary 1; Table S1). We analysed attitudes toward EO water quality data products in the light of differences in participants’ geographic origin, role in the organization and experience with satellite based EO to complete the exploration of RO3. One-way analyses of variance (ANOVA) were performed grouping participants. Two groups for testing differences in attitudes among participants with different origins were North America and Europe versus other countries. Three groups included field sampling versus program managers versus others when testing differences in the role played by participants in the organization. Two groups were experienced versus not experienced when analysing differences in attitudes according to participants’ experience with EO water quality data products. We used the one-way ANOVA with post-hoc Tukey test to determine whether there are any statistically significant differences between the means of each attitude in these groups (Kim, 2017).

When applying means, standard deviations and one way ANOVA tests, this study analyses Likert scales as interval scales. This is common in many studies in social sciences and studies analysing psychometric concepts in particular (like trust or attitudes) (Mulder et al., 2006; Lau and Lee, 1999; Carifio and Perla, 2007). Research also highlights that the application of parametric tests to ordinal variables in most cases conducts to the same results as non-parametric tests (de Winter and Dodou, 2010).

## 3. Results

A total of 171 participants responded to the survey. It was not possible to track the exact number of individuals the survey initially reached because it was distributed through the AquaWatch list serve, associated organizational emailing lists, and advertised on social media and websites, however we estimate ~12 thousand survey recipients/views. Forty (40) individual responses were excluded due to lack of completion, resulting in 131 surveys used in statistical analysis. The sample size is comparable with previous studies analysing technology adoption by professionals (Ahmad et al., 2019; Kamble et al., 2019; Knoche and Hasselbring, 2019).

Of all participants in this globally distributed survey, the majority are from the Americas (Table 2). Nearly half of the participants indicated that they work in academia, followed by more than one third in government. Water quality monitoring is actively conducted by scientists from the government, private industry (contractors), and academic sectors, with responses from either equally representative of water quality expertise. Within these organizations, nearly one third of participants serves as program managers or work in field sampling roles. In terms of thematic focus areas within water resources and water quality, over half of participants indicated that they work in ambient surface water monitoring of inland and coastal water bodies and more than one fifth in EO. Yet respondents of those sectors may vary in knowledge of legal mandates or water quality parameter limits.

### 3.1. Participant knowledge of EO

The participants’ knowledge and understanding of satellite EO water quality data products were explored through univariate data analysis techniques, along with analysis of relative frequencies of the data to show the distribution of the analysed ordinal variables and mean and standard deviations of the distributions to analyse central tendency and homogeneity respectively. Gauging participant knowledge and experience with satellite EO technologies (Table 3), most of the participants (40.5 %) self-identified as beginners with little understanding of satellite EO. One-fifth of the sample indicated that they were experienced users and nearly 18 % expert users. Nearly 17 % declared themselves novice users, unaware and lacking any knowledge of satellite based EO technology.

#### 3.1.1. Legal reporting requirements

One of the main objectives of this water quality data survey was to poll participants’ knowledge of how their country of residence/study deals with water quality data. Specifically, whether their country is involved in monitoring water quality; whether data are available to the public, the legal reporting requirements for coastal and inland water quality monitoring at the geographical level, and the required parameters; and the involvement of their country’s citizens in monitoring water quality. A total of 47.3 % of the participants failed to respond to the survey question on how satellite EO technology contributes to national reporting requirements, and 48.1 % failed to respond to whether satellite EO contributed to resource management decisions in their country (Table 4). Almost one-third of participants indicated that satellite EO contributed to resource management decision making in their country. A total of 10.7 % of participants said that satellite EO contributed to fulfilment of national regulatory requirements.

**Fig. 2.**
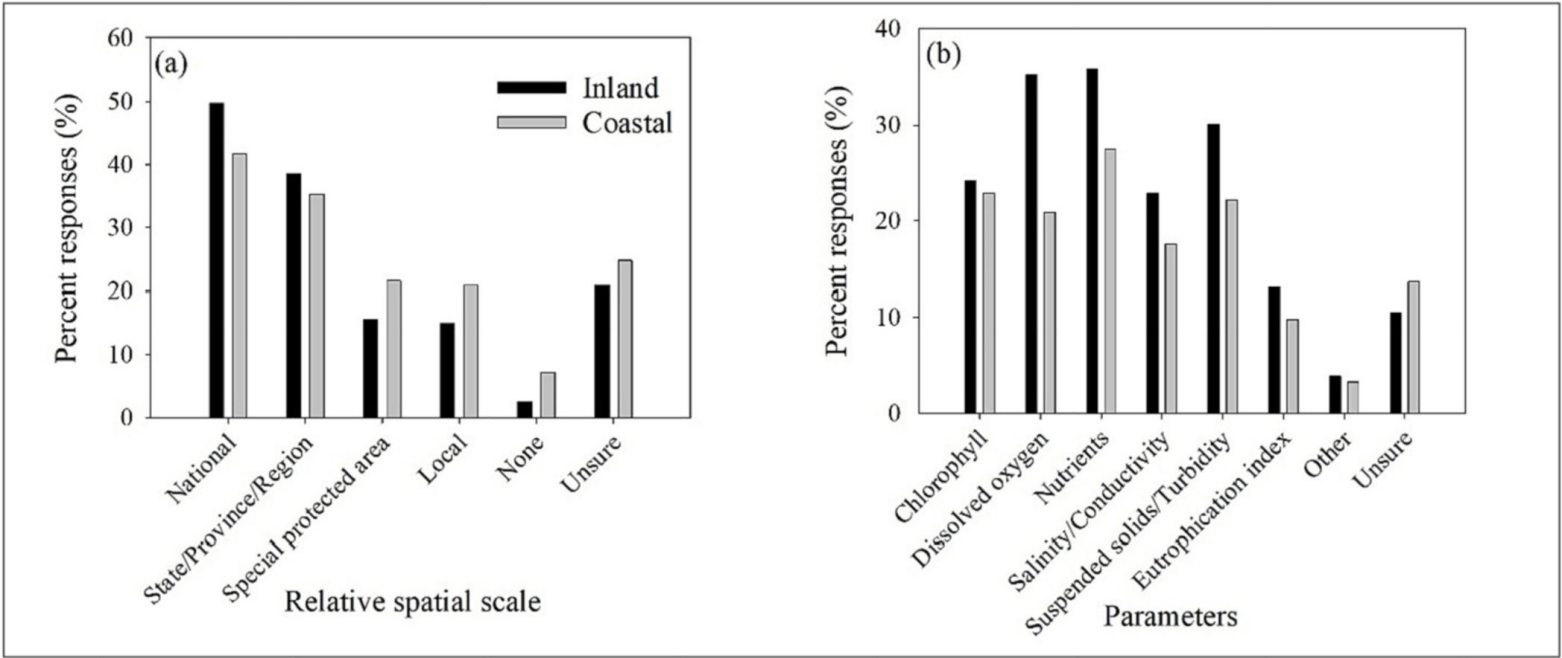
Distribution of responses within the water sector based on (a) spatial scale and (b) water quality parameters of interest for coastal and inland waters.

Participants indicated that most reporting requirements were at the national and state/province/region spatial scales (Fig. 2a), with slightly increased responses for inland over coastal waters. Special protected areas and local areas had the lowest responses for the four defined areas, but with slightly higher responses for coastal waters. Water quality parameter reporting had the greatest responses for dissolved oxygen, nutrients, and suspended solids/turbidity in inland waters (Fig. 2b). Coastal water reporting was highest for nutrients, suspended solids/turbidity, and chlorophyll. The total of all responses could exceed 100 % because participants could have answered more than one option depending on their operating and reporting requirements. In North America and Europe, 72.6 % of participants indicated that citizens are involved in water quality monitoring including collecting water samples, making field measurements, and providing photos. Citizen science and involvement in the rest of the world was significantly less (30.18 %, chi-square: 13.369, p-value: *<*0.001).

### 3.2. Problems accessing water quality data

Identification of barriers and problems in the adoption of all EO (RO2) was reached by analysing the perceived level of problem in accessing and interpreting conventional water quality data, analysing relative frequencies and means and standard deviations. The most prevalent concern the participants faced when attempting to work with water quality data involved issues around accessibility and interpretability of data (Table 5). In accessing water quality data, 30 % (30.5 %) of survey participants indicated that they experienced slight problems and 25 % (24.5 %) indicated that they experienced no problems at all. Just over one-third (34.4 %) indicated that they have no problem with interpretation of available data. However, 12.2 % of participants indicated that they have never accessed water quality data and 15.3 % had never attempted to interpret water quality data.

Many of the participants (n = 112; 86 %) indicated that a lack of data availability, particularly related to spatial location, resolution, latency, timeliness, and temporal availability, was the biggest problem they experienced with using EO water quality data for decision-making (Table 6). It is important to note that while many satellite EO are available globally, limited spatial resolution and thus lack of data availability at “desired locations”, was a major problem in using water quality data among survey respondents. A smaller percentage of participants expressed poor confidence and trust in the quality of water quality data. An even smaller percentage declares lack of understanding or knowledge in interpreting EO water quality data, despite 16.8 % recognised themselves as unaware and lacking any knowledge of satellite EO technologies (Table 3). This inconsistency may be due to a sort of hierarchy of problems: the main issues highlighted by respondents are linked to lack of availability, therefore issues related to knowledge show to fade into the background. Similarly, 41.2 % of participants echoed this lack of availability sentiment when asked about barriers in accessing water quality data. Barriers linked to technology and costs were not identified as being so relevant. Other barriers (16.8 %) elicited by participants were linked to: the lack of knowledge of the existence of data; data being stored in different databases by different entities; data only being available in poor formats; delays in data availability; difficulty in navigating websites that contain data; inconsistencies in data parameters; and the bureaucracy required to access data.

### 3.3. Attitudes toward satellite EO water quality data products

Most survey participants expressed a very positive or positive opinion about the usefulness of remotely sensed water quality data in fulfilling water quality reporting requirements in their country (Table 7). Likewise, participants indicated a high level of trust with in-situ EO and satellite EO water quality data. Based on these results, participants indicated greater levels of trust with in-situ EO water quality data than remotely sensed water quality data.

#### 3.3.1. Determinants and implications for trust in EO water quality data products

Table 8 shows results of Spearman’s correlations applied to the attitudinal variables collected through the survey. A significant and positive correlation between the trust placed in satellite EO water quality data and the perceived usefulness of this data emerges (+0.204). The usefulness of EO data is significantly and positively correlated with two attitudes related with knowledge, namely participants’ stated knowledge of satellite EO technologies (+0.293) and their perception of relevance of this data in the country where they live (+0.288). Trust in satellite EO water quality data is also significantly and positively correlated with trust in in-situ EO water quality data (+0.259), which in turn is negatively correlated with the perceived problem in accessing in-situ EO water quality data (− 0.259). No significant relationship emerges between trust in satellite EO water quality data and knowledge of these technologies or problems in accessing water quality data.

#### 3.3.2. Attitudes in the light of participants’ characteristics

Table 9 shows results from one-way ANOVA, with attitudinal variables analysed in the light of respondent’s characteristics (geographic location, role in the organization and experience with EO technologies). It shows that respondents from North America (Canada, Mexico, and the US) and Europe are more prone to think that satellite EO technologies have no contribution to their country’s national reporting requirements than people from other parts of the world (average relevance of 0.7 vs 1.1 respectively, on a scale from 0 to 2, with F = 5.69) and that these technologies are less useful for water quality data (4 vs 4.5, on a scale from 1 to 5). The same participants in North America and Europe perceived fewer problems in accessing water quality data (2.2 vs 3.3, on a scale from 1 to 5). No significant differences emerge considering knowledge and trust in satellite EO technologies.

The role of participants within their organizational structures also affects the relevance and knowledge of the satellite EO technologies. Program managers tend to declare that EO is less important in contributing to a country’s national reporting requirements than people involved in field sampling or other tasks (mean relevance of 0.5 for program managers vs 1.1 for people involved in field sampling and other tasks). However, people involved in field sampling have less knowledge of EO technologies for water quality monitoring than program managers and people with other roles in the organization (2.1 vs 2.5 and 2.7 respectively). Participants’ experience with EO technologies has an impact on knowledge of these technologies, with more experienced participants displaying a higher knowledge (3.2 vs 1.8). However, experience does not influence any of the other constructs, like relevance, usefulness, or trust.

## 4. Discussion

### 4.1. Participant knowledge and perception associated with EO water quality data

Due to most survey respondents being from North America (66 %) and within academia (45 %), these survey results represent a subset of water quality professional responses from North America, Europe, Africa, Asia, South America, Central America, and Oceania. Among the survey respondents within academia and the government/industry water management sector, satellite EO remains a novel entity, with limited appreciation of what it can deliver for the sector - less than half of survey participants responded to questions regarding how satellite EO data might be used in reporting and decision making. One-third (33.3 %) of survey participants consider EO in decision making for resource management, however it is not exactly clear whether this relates directly to water management. Similarly, 10 % (10 %) of survey participants indicated that EO contribute to national legal regulatory requirements. However, it would be useful to know the basis for which EO is being used in such a legal context and identify examples of EO data being used to support decision making and the development of digital Earth strategies.

### 4.2. Perceived relevance and usefulness of satellite EO water quality data

As a case example, the U.S. states are just beginning to take advantage of satellite data for decisions. Examples include the Wyoming Department of Environmental Quality and Utah Department of Environmental Quality using satellite EO data for issuing recreational health advisories due to cyanobacteria harmful algal blooms. Satellite data source references are starting to show up in planning and guidance documents such as the Wyoming Harmful Algal Bloom (HAB) action plan, New Jersey HAB action plan, Interstate Technology Regulatory Council (ITRC) monitoring strategies (ITRC, 2021), World Health Organization (WHO; Welker et al., 2021), and even some state laws such as the Oregon 2018 cyanotoxin drinking water rule. States may consider satellite EO data to complement field measures. Staff at state agencies have said, “the data allows [us] to better target field sampling and more efficiently use our limited resources”; and that it helps “visualize patchiness and provides additional context” (Schaeffer et al., 2019). The Water Framework Directive provides in legal framework within Europe, the requirement is to improve water quality status. Within the Scottish Environment Protection Agency (SEPA), EO is beginning to be used to assess improvements in water quality status for lakes and reservoirs. Beyond lakes and reservoirs, the obstacle to using EO for water quality is largely limited by issues of spatial and temporal resolution to assess discharges into riverine environments for compliance needs. Nevertheless, the opportunity offered by next generation spaceborne monitoring has the potential to offer compliance monitoring.

**Fig. 3.**
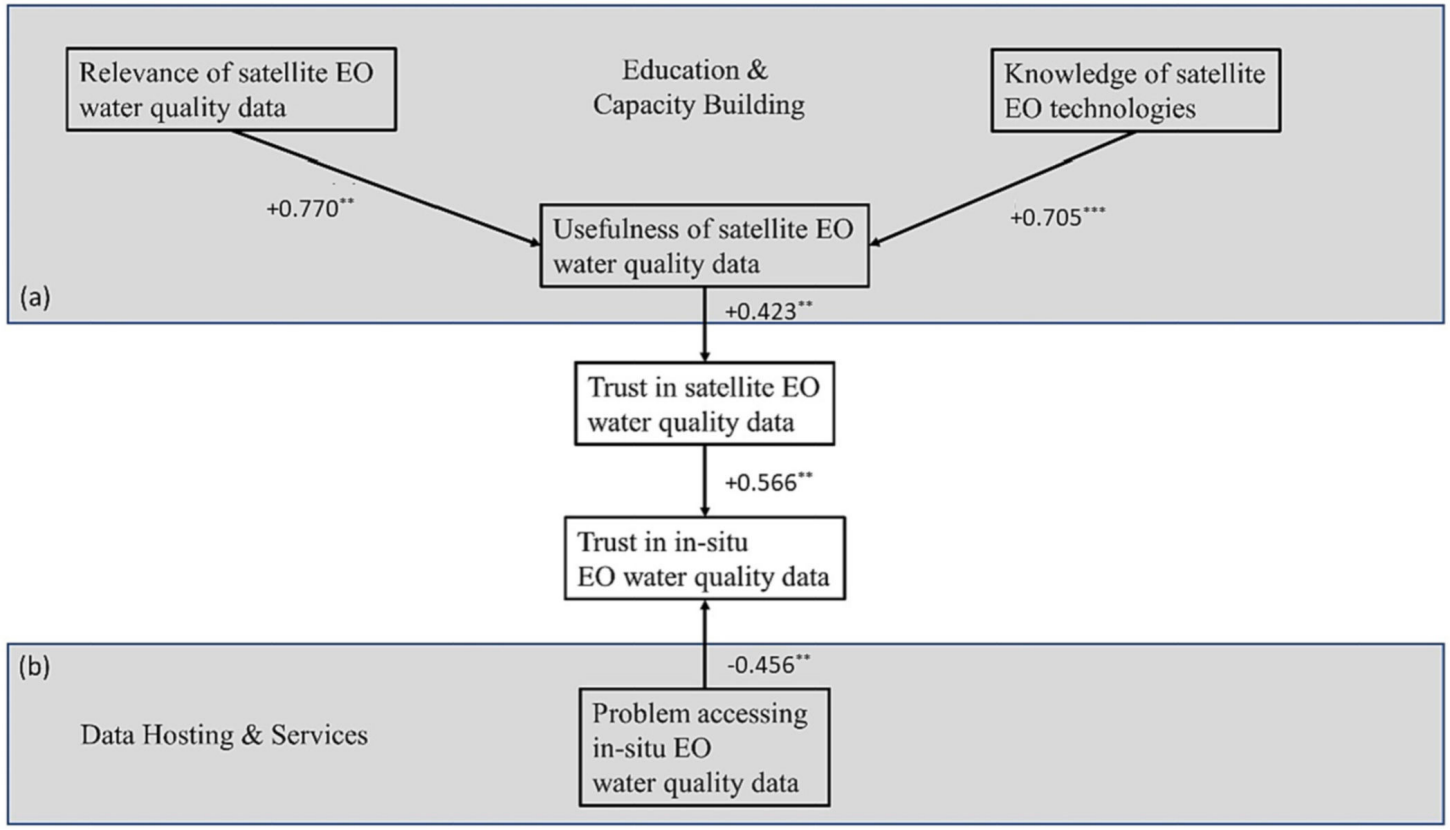
Conceptual model for empirical analysis. Box (a) indicates that users build trust with in-situ data if they have relevant satellite EO data and knowledge of how to appropriately apply the data toward their program objective. Box (a) is dependent on the satellite EO community providing education and capacity building to the water sector supporting both the knowledge of technologies and relevance to programs. Box (b) indicates that in-situ data trust is negatively impacted by limitations of access to in-situ water quality data. The condition in box (b) could be addressed through publicly accessible data hosting and services to the water sector. *** and ** denote statistical significance at the 1 % and 5 % levels, respectively.

The concept of better targeting field sampling and providing additional context supports the conceptual model (Fig. 3) from this survey of the water quality sector. Relations among the conceptual model components are tested applying ordinal logistic regressions. Fig. 3 shows regressions coefficients. Further details on the applied models and their goodness of fit are reported in Supplemental 1. The top part of the model indicates that users will build trust with in-situ data if it is supported by useful satellite EO data. This is illustrated in the previous case examples from Utah and Wyoming, where satellite data is used to inform more precise field sampling and provide a better spatial and temporal context across systems. Perceived usefulness of satellite EO water quality data was conditioned by relevance of the satellite EO data and knowledge of the technologies that fall within GEO and space agency policies for education and capacity building.

There are similar trends of adoption also occurring in other parts of the world, including Europe, Australia and South Africa (Greb et al., 2018). From a SEPA perspective, there is a need to demonstrate that the EO opportunity offers data that is equivalent or better to the sampling frameworks that it replaces. Currently, the data being used in SEPA is helping to inform sampling priorities for compliance monitoring in lakes and reservoirs. This includes public reassurance monitoring as well as testing efficacy for future legal reporting. Given the large number of lakes, currently Finland is the only European country to be using EO within its legal reporting mechanisms.

A global inventory of known water quality projects is available at GEO AquaWatch. However, there remain substantial parts of the world that are deficient in water quality data for effective water management and governance. In the same way that mobile phone technology has been adopted over and above the development of conventional landline technology, EO has the potential to leapfrog (Brezis et al., 1993) the implementation of more conventional sampling and monitoring frameworks. EO can therefore substantially benefit data poor regions, facilitating new knowledge and understanding to drive governance change and co-create solutions that collectively tackle the most fundamental global water challenges such as safe water, human health, and food security.

### 4.3. Attitudes and behavior model

From the model illustration (Fig. 3) bottom section, in-situ EO water quality data trust is negatively impacted by access limitations to the data itself. In-situ EO water quality data can be hosted through a confusing array of web resources or be altogether inaccessible. Satellite EO data access limitations may result in the same negative impacts if efforts are not supported for centralized data hosting and services. The European Union’s EO program currently provides services for six domains: atmosphere, marine, land, climate change, security, and emergency. Data and products related to water, hydrology and hydrodynamic processes are already delivered under several of these services. However, they are provided separately and for specific purposes. This disconnect in service provision between water quality and quantity are siloed by issues of geographical definition, such as inland versus coastal waters. This hinders our system understanding of the water continuum and water cycle. This siloed approach to water monitoring and assessment also contributes to issues in data trust due to limitations in data access, as demonstrated by the existing and disconnected network of data hosting and services for in-situ EO measures. Connecting satellite databases with in-situ databases would also provide significant benefits – growing and retaining confidence in the data, and broadening understanding across both datasets. The growing awareness of this requirement is feeding into the development of the next generation of Copernicus Services provision for Water through European projects such as Water-ForCE.

Less than ten years ago, Schaeffer et al. (2013) and Turner et al. (2015) demonstrated that access to satellite imagery could be limited due to financial costs of acquiring the data, which was likely a remnant of when the Landsat program charged for imagery. The shift in responses to this survey where costs were reported being less of a concern was likely supported by the widespread knowledge of the Copernicus program’s free and open data access policy (Berger et al., 2012).

### 4.4. Proposed solution: a need for an integrated approach

Without surveying the user community, we would not have identified the challenges identified in this paper. We crossed sectors of policy, industry, and various water communities to summarize the status of acceptance of this technology. The results of the survey flag the presence of a gap between research science, applied social science, and water policies. An interdisciplinary approach to studying water is emerging as a customary practice in bridging the understanding of the range of water disciplines and issues surrounding management of water. Another term, transdisciplinary, a concept that has recently been gaining traction in the water research field, extends the study of water research beyond that of various disciplines to define the research based on socially relevant issues (Barry and Born, 2013; Krueger et al., 2016; Pohl, 2011). A core facet of transdisciplinary research is coproduction of knowledge and participatory research among scientists and non-scientists. Inclusion and participation of non-scientists, stakeholders, decision-makers, and public policy in water research and governance has proven instrumental in the acceptance and trust of water policies and technologies (Krueger et al., 2016; Stirling, 2008; Tsouvalis and Waterton, 2012). Rather than merely giving non-traditional users (including indigenous peoples) a “seat at the table” of the EO/water quality conversation simply for the sake of inclusion, it is crucial that we rephrase our communal water narrative into one of deep respect, reciprocity and learning from those whose knowledge and practice of sustainability extends beyond academic water research and/or professional knowledge (Ban et al., 2018; Nelson, 2008; Ogar et al., 2020). Such a concept has been summarised as the Quintuple helix (Barth, 2011), where the research community, together with industry, government, and communities address the climate-induced challenges of the natural environment. As an example, water quality standard programs often rely on discrete and quantitative measures of the biological, chemical, and physical factors of surface water. Qualitative water user perception has emerged as an alternative measure of water quality to increase engagement with communities. Water user perception is unique because it integrates multiple environmental characteristics such as water color, transparency, odor, and biology and is also uniquely connected to the community within proximity of the water body (USEPA, 2021).

Understanding the needs, barriers, and experiences of various water sector groups related to research, monitoring, and governance is a key step in the beginning phases of user-driven research on socially relevant issues and solution-driven systems. How, and for whom, water is being governed has impacts on river flows, groundwater tables and pollution levels, affecting both upstream and downstream water users (Kattan, 2006). The capacity of countries to pursue poverty reduction strategies, integrate water resources management plans, meet new demands, and manage conflicts and risks depends on the ability to promote and put into place sound and effective governance systems.

There are sound prospects for governance research to improve the interpretative resources for natural science data, and vice-versa. This is required to promote better water allocation regimes that manage trade-offs across water users and uses, as well as stronger multi-level governance systems that better reconcile priorities and improve capacity at different levels of government. The survey results, analysis, and interpretation presented here reveal complex social and technical challenges related to water issues, Earth observations, and the relationship among users, decision-makers, and governance systems. Engagement with indigenous and native people is another opportunity, and an area of capacity development where the global EO water quality community needs to invest effort and resources to become more inclusive.

Open access to high-quality observational data enhances knowledge across this spectrum. While there are several different legitimate knowledge areas in relation to water and its use, each is subject to important critiques (Table 10).

What happens at the boundaries between the knowledge areas in Table 10 is important. A boundary is often a zone of contested space, capital, and meanings (Kearney, 1991). For many, the real challenge is coming up with appropriate solutions, tailoring them to local contexts and overcoming obstacles to reform. Failures are often caused by disregard of local factors and local actors. Yet local groups and individuals are often without access to information, are excluded from water decision making, and thus lack the capacity to act (Kattan, 2006). A key role could involve knowledge brokering and translation. Improved accessibility to EO data improves understanding with visual displays of quantified results, compensating for the various inadequacies of different knowledge forms and mitigating knowledge conflicts.

**Fig. 4.**
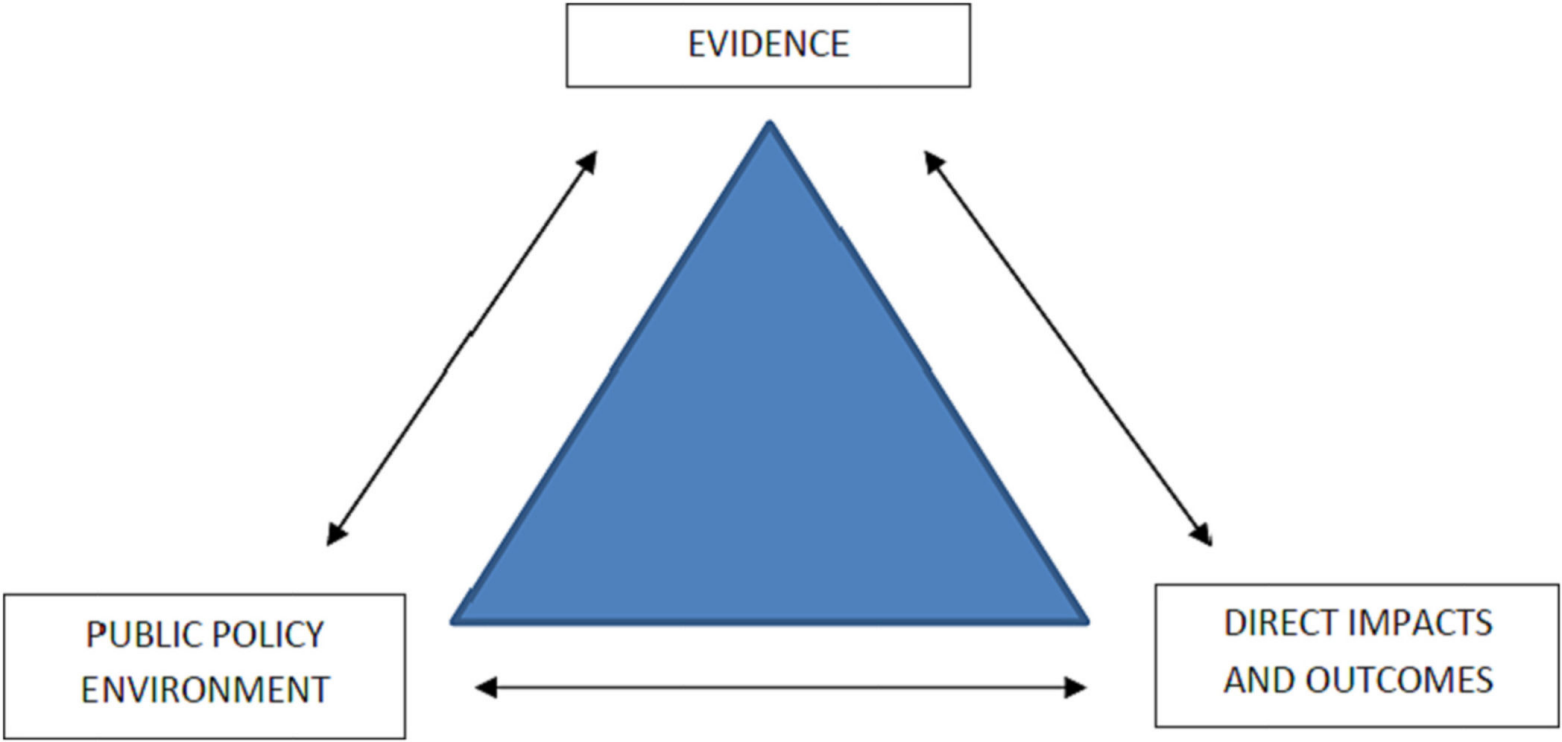
Conceptual relationship between evidence, the policy environment, and impacts relevant to inform data-driven decision making.

Moreover, important relationships may be conceptualised in the triangle between evidence, the public policy environment, and direct impacts and outcomes (Fig. 4). Evidence is required to inform both the public policy environment and stakeholders responsible for delivering direct outcomes and impacts such as food security, better sanitation and health and wellbeing. For example, in 2018, Laura Tuck, VP at the World Bank stated that, ‘EO provides an unbiased, consistent, and timely perspective that can inform data-driven decision-making. It therefore helps us to achieve our core mission at the World Bank’. Klasse (2018) makes the further point that ‘satellite EO provides historical as well as actual global information on a regular basis and can thus rapidly reveal where change has happened in a consistent repeatable and unbiased manner’. In summary, satellite EO evidence may or may not find purchase in the public policy environment. If it does, it will help create a virtuous circle if the chain is strengthened between more effective policies and better direct impacts and outcomes. Integrated knowledge management is therefore the servant of integrated water management.

### 4.5. Conceptual framework of future efforts

The global experience of travel and in-person monitoring restrictions during the COVID-19 pandemic has accelerated consideration of satellite EO technology. COVID-19 social distancing requirements may have limited environmental monitoring and research efforts, especially those monitoring activities not related to COVID-19. Anecdotal evidence from the U.S. and Europe included field teams that were hindered in monitoring efforts due to COVID social distancing requirements and travel restrictions (McGrail et al., 2020) and were assisted by satellite EO technology in the presence of these restrictions. In the years beyond the global COVID-19 pandemic this technology will continue to reduce travel and monitoring costs, and improve speed and efficiency in responding to predicting, and mitigating natural disasters. Satellite EO will also bridge data gaps in conflict areas as government structures change or break down; the data obtained will be just as useful after conflicts are resolved and governments become re-established.

Monitoring of trends and spatial patterns can reveal previously missed or new environmental issues. The three-dimensional aspect of space and time offered by satellite EO may advance the science in previously unknown environmental concepts. These potential new discoveries contribute to new research and policies that have global impact (Lovett et al., 2007). Future research may thus take further the relationship between evidence, policy and direct impact, moving beyond generating and reporting on the evidence to consider the views and perceptions of those who use this evidence. This increases the requirement for constructive technology assessment, to further ‘articulate the demand-side of technology development’ (Schot and Rip, 1997) and surface any further practical, ethical or moral dilemmas inherent in these innovations (Joss and Bellucci, 2002).

There are potential policy concepts that may limit the immediate uptake of satellite EO data for regulatory reporting and adoption of new EO technologies. A specific example of this is demonstrated in Water Framework Directive reporting of water quality (Commission of the European Communities, 2000). Here we have a classification that is based on the relationship between contemporary water quality and a historical baseline from either modelled or observed measures. We can estimate contemporary water quality using satellite EO data. However, the two-dimensional information provided by satellites does not compare to single-point sample measurements. Even in an aggregated form, such as a satellite EO water body mean statistic, these parameters are different and as such, satellite EO estimates may not be directly compared to historical baseline values. This prevents the simple substitution or inclusion of satellite EO data for reporting. In this case, baseline values may need adjustment to represent satellite EO outputs or modeling the impact of the additional spatial information on single-point samples. The decision on how to proceed, and whether existing requirements are amended or new requirements are developed specifically for satellite EO applications, will ultimately depend on long-term reliability and sustainability of satellite EO monitoring. Here, the industry has an excellent track record of comparing across platforms (Martínez-Vicente et al., 2017) long-term trends in the physical and biological characteristics of our oceans – provide a valuable record of ocean warming and primary productivity (Mélin et al., 2017 ).

Similar concepts exist under the U.S. Federal Water Pollution Control Act/Clean Water Act (CWA), a federal environmental framework intended to protect the nation’s water resources from pollution discharges. The Clean Water Act established responsibilities in the governance of the nation’s surface waters - including surface water pollution standards; limiting the discharge of pollution in accordance with those standards through permitting, ambient surface water quality monitoring and assessment; and intervening with source control and remediation actions in cases of non-compliance. These regulations involve a wide-ranging network of policies and practices that would also need to be navigated (Table 11). For example, effective integration of satellite EO would necessitate first understanding of the differences between what is generated through satellite EO and what the current/historical conventions state and federal regulatory agencies generate to implement these CWA programs. Differences include the spatial and temporal observational scales, data densities, and the duration and frequency of the information that is produced. The more challenging issues that could constrain the uptake of satellite EO for CWA purposes lie in reconciling those differences in ways that match the needs of regulatory programs, the timeframes of the regulatory cycles, and the scales of existing regulatory applications.

## 5. Conclusions

Water is central to life and the prosperity of our planet. However, the multiple competing pressures on the numerous ecosystem services that water provides is increasingly unsustainable, with those pressures compounded further by climate change. New intelligence on the dynamic nature and status of water quality of our inland water bodies is needed to promote more effective water basin management. Recent advances in satellite EO now enable consistent, unbiased monitoring of disparate water bodies and provide the data needed. However, there are still significant bottlenecks to the implementation and uptake of EO technology. Establishing a baseline of understanding across the global water sector through this survey was a critical first step for further discussion and research. In lifting the lid on these important issues, this paper provides a key contribution to these endeavors. A revised survey (s) could be undertaken annually in the future, with refined questions to resolve information gaps and illuminate understanding of sector-specific responses.

Engaging with the water sector community around the world and soliciting feedback on the use and value of EO data for water quality and water resource management is a relatively new activity within the context of water governance and the public policy space. While results suggest varied levels of understanding the opportunity satellite EO presents to the user community, there are examples of the gradual integration of satellite EO data into policy and decision making. There is growing evidence that satellite EO is also helping to guide conventional water quality sampling and build trust in its technology. The analysis and interpretation presented here enables further activities that will expand upon the initial scope of the survey, including enhancements to the technology’s social and economic value and increased capacity in EO science and implementation. Eventually, universal access to the data derived from satellite EO will break down information barriers and provide an opportunity to broker knowledge across sectors, communities, geographical, and political boundaries. In this way, the common goal of delivering safe water for all can begin to be achieved. Policy translations of this technology remain, given current disconnects between policy and existing capabilities. While the satellite EO capability is clear, work is still required to resolve disconnects between technology and policy to ensure compatibility between conventional and new EO approaches. These bottlenecks are not permanent, and in a post-COVID-19 pandemic world, there will be real opportunities to design the next generation of satellite platforms to meet the operational monitoring requirements of the global water sector and deliver continuous improvements in monitoring and understanding of water quality.

## Supplementary Material

Supplement1

## Figures and Tables

**Table 1:** Information collected through the survey.

Topic	Type	Categories

*1) Participant characteristics*		
Country	Nominal	All different countries in the world
Sector	Nominal	Government, academia, non-profit, private sector, other
Interest in water quality	Nominal	Drinking water monitoring, ground water monitoring, in-situ ambient surface water monitoring of inland and coastal water bodies, satellite-based EO, wastewater monitoring, other
Role	Nominal	Field sampling, laboratory analysis, program manager, other
*2) Participant knowledge*		
The country is involved in monitoring water quality	Binary	0 = no, 1 = yes
Data are available to the public	Binary	0 = no, 1 = yes
Legal reporting requirements in coastal and in inland water quality monitoring at geographical level	Nominal	Local, special protected area, state or province or region, national, none, unsure
Legal reporting requirements in coastal and inland water quality monitoring at parameters level	Nominal	Chlorophyll a, Dissolved Oxygen, Nutrients, Salinity/Conductivity, Total Suspended Solids/Turbidity, Eutrophication Index, other, unsure what parameter but something required
Involvement of citizens	Binary	0 = no, 1 = yes
Role of citizens	Nominal	Collecting water samples, making field measurements, providing photos, other
*3) Attitudes*		
Relevance of satellite remote sensing water quality data	Ordinal	0 = no rule of satellite based EO in contributing to national reporting requirements, 1 = voluntary role, 2 = fulfills legal requirements
Knowledge of satellite EO technologies	Ordinal	1 = unaware and lacking any knowledge of satellite EO, 2 = a beginner with little understanding, 3 = experienced but would welcome more training, 4 = an expert
Usefulness of satellite EO water quality data	Ordinal	From 1 = not useful at all to 5 = very useful
Trust in satellite EO water quality data	Ordinal	From 1 = not at all trustworthy to 5 = very trustworthy
Trust in in-situ EO water quality data	Ordinal	From 1 = not at all trustworthy to 5 = very trustworthy
Problems accessing water quality data	Ordinal	From 1 = no problem to 5 = no access at all
Problems interpreting water quality data	Ordinal	From 1 = no problem to 5 = no access at all
*4) Barriers and problems*		
Problems in using water quality data	Nominal	Not available for the desired time, Not available for the desired location, Not available quickly enough to be useful in decision-making, Poor quality or not trustworthy, Not understand or know how to interpret the available in-situ data, Not understand or know how to interpret the available EO data
Barriers in accessing water quality data	Nominal	Data not available, I need a better computer or technology, I must pay a large fee, I need a login or password, Other

**Table 2:** Sample characteristics (n = 131).

Characteristics	n	%

Continent		
North America	66	50.4
South America	9	6.9
Europe	22	16.8
Asia	16	12.2
Africa	16	12.2
Oceania	2	1.5
Organization sector		
Academia	59	45.0
Government	49	37.4
Non-profit	12	9.2
Private sector	8	6.1
Other	3	2.3
Role in the organization		
Program manager	42	32.1
Field sampling	40	30.5
Laboratory analysis	13	9.9
Other	36	27.5
Primary interest in water quality		
In-situ ambient surface water monitoring of inland and coastal water bodies	66	50.4
EO	37	28.2
Ground water monitoring	15	11.5
Drinking water monitoring	11	8.4
Wastewater monitoring	2	1.5

**Table 3:** Knowledge of EO technologies (n = 131).

	Unaware and lacking any knowledge (%)	A beginner, with little understanding (%)	Experienced, but would welcome more training (%)	An expert (%)	Mean	SD

Knowledge of satellite EO technologies	16.8	40.5	25.2	17.6	2.4	1.0

Note: Knowledge was based on a skill from 1 through 4, with 1 = unaware and no knowledge and 4 = expert. SD = standard deviation.

**Table 4:** Contribution of satellite EO for water quality requirement and resource management decisions in the country.

	N	%

Contribution of satellite EO toward resource management decisions in the country		
Yes	39	29.8
No	30	22.8
Relevance of satellite EO water quality data to the country’s national reporting requirements		
No contribution	24	18.3
Only voluntary reporting	30	22.9
It fulfills legal requirements	14	10.7

**Table 5:** Problem perception for water quality data access and interpretation (n = 131).

	1 - No problem (%)	2 - Slight problem (%)	3 - Moderate problem (%)	4 - Large problem (%)	5 - Not accessed/interpreted (%)	Mean	SD

Problem accessing water quality data	25.2	30.5	19.8	12.2	12.2	2.6	1.3
Problem interpreting water quality data	34.4	26.0	19.1	5.3	15.3	2.4	1.4

**Table 6:** Problems in using and barriers to accessing water quality data for decision-making.

Problems and barriers (participants could check more than one response)	n	%

Problems in using water quality data		
Data is not available for the desired location	42	32.1
Data is not available quickly enough to be useful in decision-making	37	28.2
Data is not available for the desired time	33	25.2
Data is poor quality or not trustworthy	21	16.0
Don’t understand or know how to interpret the available in-situ water quality data	4	3.1
Don’t understand or know how to interpret the available EO water quality data	2	1.5
Barriers to accessing water quality data		
Data I seek is not available	54	41.2
I need a better computer or technology to view the data I seek	13	9.9
I must pay a large fee for the data I seek	14	10.7
I need a login or password to access the data I seek	23	17.6
Other	22	16.8

**Table 7:** Usefulness and trust in satellite EO water quality data products (n = 131).

	1 - Not (%)	2 (%)	3 (%)	4 (%)	5 - Very much (%)	Mean	SD

Usefulness of EO in fulfilling water quality reporting requirements in the country	0.9	8.2	11.8	35.5	43.6	4.1	1.0
Trust in in-situ water quality data	0.0	5.6	8.3	57.4	28.7	4.1	0.8
Trust in available EO water quality data products	1.8	9.9	31.5	49.5	7.2	3.5	0.8

**Table 8:** Spearman’s correlations matrix, attitudinal variables.

Attitudes	2.	3.	4.	5.	6.

1. Relevance of satellite EO water quality data	0.240	−0.042	−0.066	0.288**	0.270**
2. Trust in satellite EO water quality data		0.208**	0.159	0.204**	−0.107
3. Trust in in-situ water quality data			0.037	0.015	−0.259***
4. Knowledge of satellite EO technologies				0.293***	−0.025
5. Usefulness of satellite EO water quality data					0.198
6. Problem accessing EO water quality data					

**Table 9:** Differences in attitudes according to adopters’ characteristics.

Attitudes	Geographic location				Role in the organization					Experience in EO			
	North America & Europe	ROW	F	Sig.	Field sampling	Program managers	Other	F	Sig.	Not experienced	Experienced	F	Sig.

Relevance of satellite EO water quality data	0.7	1.1	5.690	0.020	1.1^a^	0.5^b^	1.1^a^	6.311	0.003	–	–	–	–
Knowledge of satellite EO technologies	–	–	–	–	2.1^a^	2.5^b^	2.7^b^	4.572	0.012	1.8	3.2	139.783	0.000
Usefulness of satellite EO water quality data	4.0	4.5	6.839	0.010	–	–	–	–	–	–	–	–	–
Trust in satellite EO water quality data	–	–	–	–	–	–	–	–	–	–	–	–	–
Trust in in-situ water quality data	–	–	–	–	–	–	–	–	–	–	–	–	–
Problem accessing water quality data	2.2	3.3	28.274	<0.001	–	–	–	–	–	–	–	–	–

– = not significantly different.

ROW = Rest of the world.

Means with different letters within a row are significantly different from each other applying Tukey test (p-value < 0.05).

**Table 10:** Competing/complementary forms of knowledge.

Form of knowledge	Critique

Individual (e.g., Water consumer)	‘Biased’
Local (e.g., Local community)	‘Anecdotal’
Specialized (e.g., Water services professional)	‘Inaccessible’
Strategic (e.g., Head of government department)	‘Disconnected’
Holistic (e.g., Academic professor)	‘Abstract’

Source: Simmons and Brennan (2016).

**Table 11:** Examples of regulatory activities that rely upon environmental observations of water quality under the U.S. Clean Water Act (33 U.S.C. §§ 1251–1388).

Regulatory activity/context	Primary authority (Oversight authority)	Statutory provisions (implementing regulations)	Timeframe	Federal guidance/policies

Water quality standards	States (U.S. EPA)	U.S.C. 1313(c) (40 C.F.R. § 131.10, 11)	3-Year cycle	(EPA, 2001)
Pollutant permitting	States (delegated) (U.S. EPA)	U.S.C. 1342 (40 C.F.R. § 122.44(d), 48)	5-Year cycle	(EPA, 2010)
Water quality monitoring	States (U.S. EPA)	U.S.C. 1256(e)(1) (40 C.F.R. § 130.4)	Annually	(EPA, 2013b)
Water quality assessments	States (U.S. EPA)	U.S.C. 1313(d) (40 C.F.R. § 130.7(b))	2-Year cycle	(EPA, 2013a)
Water quality remediation	States (U.S. EPA)	U.S.C. 1313(d) (40 C.F.R. § 131.7(c))	9–13 years	(EPA, 1999, 2018)

## Data Availability

Data will be made available on request.
