# Supplementary material for: Perspectives on user engagement of satellite Earth observation for water quality management
Source: Technol Forecast Soc Change. Author manuscript; Available in PMC 2024 Jul 17. (PMC11252903; doi:10.1016/j.techfore.2023.122357)
Supplement: Supplement1 [file NIHMS1880029-supplement-Supplement1.docx]

**Supplementary Material 1**

Ordinal Logistic Regressions are applied to test relationships among model components. The first and the second regression show that user perception of usefulness of satellite EO water quality data is positively influenced by perceived relevance (coefficient +0.770) and knowledge of satellite EO technologies (+0.705) respectively. The third regression shows that trust in satellite EO water quality data is positively influenced by perceived usefulness of satellite EO water quality data (+0.423). The fourth and the fifth regression show that trust in in-situ EO water quality data is positively influenced by trust in satellite derived water quality data (+0.566) and negatively influenced the concerns in accessing water quality data (-0.456). Odd ratios show the odds for a unit increase in the dependent variable when changing levels of the independent variables. Odds ratios higher than 1 indicate a positive relation among variables, odds ratios lower than one indicate a negative relation. Likelihood ratio chi-square (LR Chi2) tests prove that there is a significant improvement in fit with the analyzed models relative to the intercept only model. Pseudo R2s measure goodness of fit of the models.

**Table S1.** Ordinal logistic regression coefficients, odd ratios and goodness of fits testing relations among attitudinal variables.

| Relation among attitudes | Coeff. | | Odds Ratio | | LR Chi2 | | Pseudo R2 |
| --- | --- | --- | --- | --- | --- | --- | --- |
| Relevance of satellite EO water quality data à Usefulness of satellite EO water quality data | 0.770  (0.355) | ^**^ | 2.159  (0.767) | ^**^ | 5.02 | ^**^ | 0.035 |
| Knowledge of satellite EO technologies à Usefulness of satellite EO water quality data | 0.705  (0.239) | ^***^ | 2.025  (0.484) | ^***^ | 9.25 | ^***^ | 0.035 |
| Usefulness of satellite EO water quality data à Trust in satellite EO water quality data | 0.423  (0.187) | ^**^ | 1.527  (0.286) | ^**^ | 5.18 | ^**^ | 0.020 |
| Trust in satellite EO water quality data à Trust in in-situ water quality data | 0.566  (0.265) | ^**^ | 1.762  (0.467) | ^**^ | 4.69 | ^**^ | 0.025 |
| Problem accessing water quality data à Trust in in-situ water quality data | -0.456  (0.167) | ^***^ | 0.634  (0.106) | ^***^ | 7.80 | ^***^ | 0.035 |

Note: *** and ** denote statistical significance at the 1% and 5% levels, respectively. Standard Errors among brackets
